# Cost-effectiveness analysis of a low-cost bubble CPAP device in providing ventilatory support for neonates in Malawi – a preliminary report

**DOI:** 10.1186/s12887-014-0288-1

**Published:** 2014-11-25

**Authors:** Ariel Chen, Ashish A Deshmukh, Rebecca Richards-Kortum, Elizabeth Molyneux, Kondwani Kawaza, Scott B Cantor

**Affiliations:** Institute for Global Health Technologies, Rice University, Houston, Texas USA; Department of Health Services Research, The University of Texas MD Anderson Cancer Center, Houston, Texas USA; Cancer Prevention Training Research Program, The University of Texas MD Anderson Cancer Center, Houston, Texas USA; Department of Bioengineering, Rice University, Houston, Texas USA; Department of Pediatrics, College of Medicine, Queen Elizabeth Central Hospital, Blantyre, Malawi

**Keywords:** Cost-effectiveness analysis, Neonate, Malawi, Prematurity, Respiratory distress syndrome, Sepsis, Ventilatory support, Bubble continuous positive airway pressure

## Abstract

**Background:**

A low-cost bubble continuous positive airway pressure (bCPAP) device has been shown to be an excellent clinical alternative to nasal oxygen for the care of neonates with respiratory difficulty. However, the delivery of bCPAP requires more resources than the current routine care using nasal oxygen. We performed an economic evaluation to determine the cost-effectiveness of a low-cost bCPAP device in providing ventilatory support for neonates in Malawi.

**Methods:**

We used patient-level clinical data from a previously published non-randomized controlled study. Economic data were based on the purchase price of supplies and equipment, adjusted for shelf life, as well as hospital cost data from the World Health Organization. Costs and benefits were discounted at 3%. The outcomes were measured in terms of cost, discounted life expectancy, cost/life year gained and net benefits of using bCPAP or nasal oxygen. The incremental cost-effectiveness ratio and incremental net benefits determined the value of one intervention compared to the other. Subgroup analysis on several parameters (birth weight categories, diagnosis of respiratory distress syndrome, and comorbidity of sepsis) was conducted to evaluate the effect of these parameters on the cost-effectiveness.

**Results:**

Nasal oxygen therapy was less costly (US$29.29) than the low-cost bCPAP device ($57.78). Incremental effectiveness associated with bCPAP was 6.78 life years (LYs). In the base case analysis, the incremental cost-effectiveness ratio for bCPAP relative to nasal oxygen therapy was determined to be $4.20 (95% confidence interval, US$2.29–US$16.67) per LY gained. The results were highly sensitive for all tested subgroups, particularly for neonates with birth weight 1– < 1.5 kg, respiratory distress syndrome, or comorbidity of sepsis; these subgroups had a higher probability that bCPAP would be cost effective.

**Conclusion:**

The bCPAP is a highly cost-effective strategy in providing ventilatory support for neonates in Malawi.

## Background

Forty-one percent of all deaths of children under the age of five years occur during the neonatal period, i.e., within the first 28 days of life [1]. Conditions that compromise respiratory function, including prematurity, birth asphyxia, and pneumonia, are responsible for more than half of the 3.6 million neonatal deaths that occur around the world each year [2]. In developed countries, mechanical ventilation, surfactant therapy, and bubble continuous positive airway pressure (bCPAP) are the major technologies used to reduce neonatal mortality from respiratory distress [3]. Due to inaccessibility of equipment and cost constraints, the developing world is in need of appropriate treatments for providing ventilatory support for neonates.

Malawi, a small landlocked country in the southeastern area of the African continent, has the highest rate of preterm births in the world: 18.1% of all newborns in Malawi are born prematurely [4]. In addition, Malawi has a neonatal mortality rate of 30 per 1000 live births [5]. The current standard of care in Malawi for babies with any type of respiratory difficulty is nasal oxygen therapy. Although bCPAP devices have been successfully implemented in low-resource settings, these life-saving machines are commercially available for approximately US$6000, and are prohibitively expensive [6,7].

In the early 2010s, a team of Rice University bioengineers and Texas Children’s Hospital physicians and respiratory therapists developed a low-cost bCPAP device that can be assembled at a cost of $350. This device delivers pressure and air flow equivalent to the bCPAP systems used in the developed world [6]. The low-cost bCPAP device has been determined to be highly efficacious compared to nasal oxygen therapy (from this point forward, bCPAP refers to this specific low-cost bCPAP). Absolute improvement in survival among neonates receiving bCPAP in one study was 27% [8]; however, the resource consumption in the delivery of bCPAP and its cost-effectiveness relative to nasal oxygen therapy was undetermined.

Using the healthcare system perspective, we sought to determine the cost-effectiveness of low-cost bCPAP in providing ventilator support for neonates in Malawi. The purpose of this study is to inform decision makers such as Malawi’s government and the World Health Organization (WHO) of the relative clinical and economic value of bCPAP compared to nasal oxygen therapy.

## Methods

We used the net benefit regression approach to perform a cost-effectiveness analysis comparing two interventions—nasal oxygen and bCPAP—targeted to treat neonates with respiratory difficulty. The overall outcomes are reported using incremental cost-effectiveness ratio (ICER) and incremental net benefit (INB).

The net benefit regression approach was deemed suitable for this study, as it accounts for individual-level variation and addresses the important issues associated with negative ICER when conducting an economic evaluation using data from a clinical trial [9,10]. The issues with negative ICER are of particular importance, as the original study outcomes were reported in terms of life and death (i.e., 1 if a patient survived and 0 if a patient died).

### Clinical study and data

To perform the economic evaluation, we used the individual-level clinical and cost data available from a non-randomized controlled study [8]. The trial was conducted over a 10-month period (from January 2012 to October 2012) in the neonatal ward of Queen Elizabeth Central Hospital in Blantyre, Malawi, in 2012. The inclusion criteria for the study were neonates with: (1) severe respiratory distress from any cause, (2) spontaneous breathing, (3) a minimum weight of 1 kg, and (4) neurological viability. Patient diagnoses included RDS, transient tachypnea of the newborn, birth asphyxia, and meconium aspiration. Clinical signs and symptoms were used to determine if comorbidity of sepsis was also present. Based on machine and staff availability, the eligible patients were given bCPAP treatment. If the bCPAP machine or appropriate staff were unavailable, the patients received standard nasal oxygen therapy and became the controls for the study. Babies who began treatment with standard nasal oxygen therapy were switched to the bCPAP machine if it became available. In the study nine infants who initially received nasal oxygen were transitioned from oxygen to bCPAP when a bCPAP device became available. The outcomes of these nine children were analyzed with the bCPAP group. 60-day survival rates and sample sizes for all patients and subgroups identified in the previous study as having an impact on survival are shown in Table 1 [8].

**Table 1:** **60-day survival rates and sizes of subgroups of neonates receiving nasal oxygen or bCPAP** [8]

**Group/subgroup**	**60-day survival**	
	**Nasal oxygen**	**bCPAP**
	**n (%)**	**n (%)**
All	11/25 (44 · 0)	44/62 (71 · 0)
Birth weight		
1.0– < 1 · 5 kg	2/13 (15 · 4)	19/29 (65 · 5)
1 · 5– < 2 · 5 kg	5/7 (71 · 4)	16/24 (66 · 7)
≥2 · 5 kg	4/5 (80 · 0)	9/9 (100 · 0)
RDS	4/17 (23 · 5)	31/48 (64 · 6)
Sepsis	0/7 (0 · 0)	16/26 (61 · 5)

bCPAP = bubble continuous positive airway pressure. RDS = respiratory distress syndrome.

### Clinical outcome and effectiveness

Neonates enrolled in the study had a clinical outcome of “died” or “discharged”. If a patient was discharged, the baby was assumed to have remained alive by day 60. All neonates were hospitalized for less than 60 days. For the lifetime analysis, if the patient died, then the discounted life expectancy of the patient was assumed to be zero years. If the assumed 60-day outcome was alive, then we assumed that the patient then had the standard life expectancy for a Malawian person of the same sex using the standard Malawian life tables for men and women in 2011 from the World Health Organization Global Health Observatory Data Repository [11].

The effectiveness used for the lifetime analysis was the standard life expectancy discounted at a rate of 3% per year for men and women [12]. The standard life expectancies of Malawian men and women are 56.80 and 58.50 years, respectively. The discounted life expectancy was estimated using a Markov model that used age-specific annual mortality probabilities from the WHO life table for Malawi for the year 2011 and a discount rate of 3%. Assuming that a newborn survives the first 60 days of life, the Markov model extrapolated the life expectancy (*D*_*LE*_) discounted for the period after the first 60 days and up to the expected time of death using the equation ${D}_{LE}\kern0.5em \approx \kern0.5em {\displaystyle {\sum}_{i=0}^{110}\frac{\left[\left(1-{m}_0\right)\left(1-{m}_1\right)\dots \left(1-{m}_{i-1}\right)\times {m}_i\right]\left(i+\frac{1}{2}\right)}{{\left(1+r\right)}^i}}$, where *m*_*i*_ is the mortality probability in the *i*^*th*^ year and *r* is the discount rate. The calculated discounted life expectancies of men and women were calculated to be 25.06 and 25.34 years, respectively.

During the clinical study, only mild and temporary complications from bCPAP treatment were observed, including nasal irritation, facial irritation, and epistaxis. Since no major morbidity was observed from bCPAP, outcomes were reported in life years (LYs) rather than the standard unit of cost-effectiveness analysis, disability-adjusted life years (DALYs) [11]. Our estimations were based on the assumption that nasal oxygen and bCPAP treatment do not decrease subsequent quality of life and survival time of infants.

### Costs

The total number of days in the neonatal ward and the level of care the infant received each day of hospitalization (i.e. nasal oxygen, bCPAP, or no respiratory support) was recorded in the clinical study. With the provided data, individual-level costs were calculated based on the level of care each day

The costs associated with spending a day with respiratory support were divided into general hospital costs and costs associated with the treatment of bCPAP or nasal oxygen. The costs associated with spending a day without respiratory support only included general hospitalization costs. For the general hospitalization cost, we applied the WHO “choosing interventions that are cost-effective” project (WHO-CHOICE) Malawi cost estimates for inpatient unit costs of a tertiary-level hospital bed-day for a day with respiratory support and a secondary-level hospital bed-day for a day without respiratory support [13]. The cost per bed-day estimates include “hotel” components of hospitalization, such as personnel, capital, and food, but not the cost of drugs [13]. The general hospitalization costs were converted to 2012 US dollars using the Consumer Price Index for All Urban Consumers: Medical Care Services as shown in Table 2 [14]. Costs associated with the treatment of bCPAP or nasal oxygen were categorized either as per-day or per-patient costs. Per-patient costs were costs that were constant for each patient regardless of the duration of hospitalization. The equipment per-day costs were calculated using a formula that annualized the costs of capital investments:

**Table 2:** **Cost estimates per patient in 2012 US$**

**Item assessed**	**Cost (US$)**	**Treatment**	**Calculation**	**Source**
Equipment				
bCPAP	350 · 00	bCPAP	Per day	Equipment vendor
Oxygen concentrator	1,248 · 00	Both	Per day	Equipment vendor
Suction machine	282 · 00	Both	Per day	Equipment vendor
Nasal prongs	8 · 43	bCPAP	Per patient	Hospital supplier
Stockinette hat	0 · 15	bCPAP	Per patient	Hospital supplier
Suction tube	0 · 56	Both	Per day	Hospital supplier
Hospital bed-day				
With respiratory support	2 · 55*	Both	Per day	WHO-CHOICE
Without respiratory support	1 · 98*	Both	Per day	WHO-CHOICE

bCPAP = bubble continuous positive airway pressure. WHO-CHOICE = World Health Organization “choosing interventions that are cost-effective” project.

*Prices were inflated from 2008 to 2012 US$ using the Consumer Price Index for All Urban Consumers: Medical Care Services for the relevant years.

$$ E=K{\left(\frac{1-{\left(1+r\right)}^{-n}}{r}\right)}^{-1}, $$

where *E* is the equivalent cost per period, *K* is the purchase price, *r* is the period interest or discount rate, and *n* is the useful life of the equipment [12]. The equipment prices were obtained from the purchase price as given by the equipment vendor or hospital supplier, adjusted for shelf life. These costs were already valued at 2012 US dollars. For all equipment, an annual discount rate of 3% was applied according to WHO guidelines [12]. The per-day costs of each piece of equipment were calculated on the basis of the annualized cost of each piece of equipment, the number of patients it served in one day, and an assumed 80% usage rate of capacity [12].

Although we recognized that providing bCPAP is more time-intensive for the nurses than providing nasal oxygen therapy, the difference in labor cost between bCPAP and nasal oxygen therapy was not included for two reasons. First, we assumed that, due to the constraints of Malawi’s healthcare system, Malawi would not hire more nurses to accommodate this extra labor requirement for bCPAP. Second, personnel costs were already accounted for in the WHO-CHOICE estimates and we did not want to count any costs twice. We also did not include training costs in our analysis. Based on the guidelines for economic evaluation, we conducted the analysis assuming that the clinicians already possessed the skills to administer bCPAP therapy and would not require further training [12]. In addition, the fixed costs associated with training would be distributed over a large number of patients, thus, rendering such costs to be negligible.

### Analysis

The results of the economic evaluation for overall outcomes were expressed in the form of ICER and INB. The outcomes for the subgroups—birth weight categories, diagnosis of respiratory distress syndrome (RDS), and comorbidity of sepsis—were reported using INB. The INB measures the value of extra patient outcome with respect to extra cost [9]. The ICER is computed as the ratio of the difference in costs and difference in effectiveness (in this study, life years).

The net benefit approach is based on the principle that a decision-maker will consider an intervention worthwhile if its cost-effectiveness ratio is less than the maximum willingness to pay per life year gained (λ). The net monetary benefit (NMB) was calculated for each patient based on λ and the incremental cost and incremental effectiveness. To conduct the analysis the data were fitted in a linear regression model using an indicator variable based on the treatment received (bCPAP versus nasal oxygen) [15].

The joint uncertainty in cost and effectiveness of bCPAP compared to nasal oxygen therapy is presented in the form of a cost-effectiveness acceptability curve [16–18]. The x-axis of the curve gives λ and the y-axis gives the proportion of estimated joint uncertainty that falls in the cost-effective half of the plane, i.e., the probability that the intervention is cost-effective.

### Protection of human subjects

The clinical component of the study was approved by the Malawi College of Medicine research and ethics committee and the institutional review boards at Rice University and Baylor College of Medicine. The economic component of the study was approved by the institutional review board at The University of Texas MD Anderson Cancer Center.

## Results

As in the original clinical trial, 87 patients were used for this study: 62 were bCPAP patients, and 25 were nasal oxygen patients. The mean number of days in the hospital of a nasal oxygen patient and a bCPAP patient were 9.1 and 15.3, respectively. From the number of days in the hospital and the level of care received each day, the average cost per patient was US$29.29 (standard deviation [SD] =26.52) for a patient on nasal oxygen and $57.78 (SD = US$40.92) for a patient on bCPAP. The effectiveness of nasal oxygen and bCPAP was 11.08 LYs (SD =12.76) and 17.86 LYs (SD =11.51), respectively. Thus, the ICER for the bCPAP intervention in comparison to the usual treatment of nasal oxygen is US$4.20 (95% confidence interval, US$2.29–US$16.67) per LY gained.

Table 3 summarizes the overall INB as well as the INB for the subgroups at a λ ranging from US$0 to US$20. At the λ = US$5, the intervention was cost-effective overall. It also was cost-effective for the subgroups of birth weight of 1– < 1.5 kg, birth weight of ≥2.5 kg, diagnosis of RDS, and comorbidity of sepsis. At λ = $20, the intervention was highly cost-effective overall and for all subgroups, except the subgroup of birth weight of 1.5– < 2.5 kg (for which the INB was negative).

**Table 3:** **INB from net benefit regression models at selected levels of willingness to pay per LY gained (λ) in 2012 US$**

**Group/subgroup**	**Incremental net benefit**				
	**λ =0**	**λ =5**	**λ =10**	**λ =15**	**λ =20**
	**(CI 95%)**	**(CI 95%)**	**(CI 95%)**	**(CI 95%)**	**(CI 95%)**
All patients	−28 · 49	5 · 38	39 · 26	73 · 13	107 · 00
	(−46 · 1–10 · 86)	(−18 · 64–29 · 41)	(−9 · 83–88 · 34)	(−3 · 08–149 · 33)	(3 · 21–210 · 79)
Birth weight					
1– < 1 · 5 kg	−39 · 71	23 · 48	86 · 67	149 · 86	213 · 05
	(−70 · 02–9 · 40)	(−2 · 91–49 · 87)	(27 · 90–145 · 44)	(53 · 99–245 · 73)	(79 · 23–346 · 88)
1 · 5– < 2 · 5 kg	−27 · 25	−33 · 58	−39 · 91	−46 · 25	−52 · 58
	(−51 · 79–2 · 70)	(−72 · 15–5 · 00)	(−129 · 72–49 · 90)	(−188 · 85–96 · 36)	(−248 · 28–143 · 13)
≥ 2 · 5 kg	−3 · 71	21 · 38	46 · 48	71 · 57	96 · 66
	(−33 · 68–26 · 27)	(−42 · 88–85 · 64)	(−55 · 85–148 · 80)	(−69 · 58–212 · 71)	(−83 · 57–276 · 89)
Diagnosis of RDS					
Yes	−33 · 70	17 · 88	69 · 45	121 · 02	172 · 59
	(−56 · 61–10 · 78)	(−6 · 08–41 · 84)	(15 · 84–123 · 05)	(34 · 86–207 · 18)	(53 · 33–291 · 86)
No	−13 · 46	−6 · 80	−0 · 14	6 · 53	13 · 19
	(−36 · 87–9 · 95)	(−54 · 04–40 · 44)	(−79 · 82–79 · 55)	(−107 · 06–120 · 12)	(−134 · 76–161 · 14)
Comorbidity of sepsis					
Yes	−26 · 64	52 · 52	131 · 67	210 · 82	289 · 97
	(−69 · 43–16 · 15)	(12 · 52–92 · 51)	(54 · 14–209 · 19)	(88 · 08–333 · 56)	(120 · 38–459 · 56)
No	−27 · 95	−7 · 81	12 · 33	32 · 47	52 · 61
	(−44 · 20–11 · 69)	(−34 · 63–19 · 01)	(−45 · 78–70 · 44)	(−58 · 29–123 · 23)	(−71 · 08–176 · 30)

INB = incremental net benefit. CI, confidence interval. LY, life year. RDS, respiratory distress syndrome.

**Figure 1:**
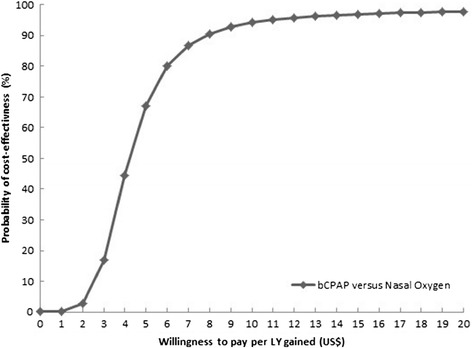
**Overall cost-effectiveness acceptability curve for bCPAP compared to nasal oxygen.**

**Figure 2:**
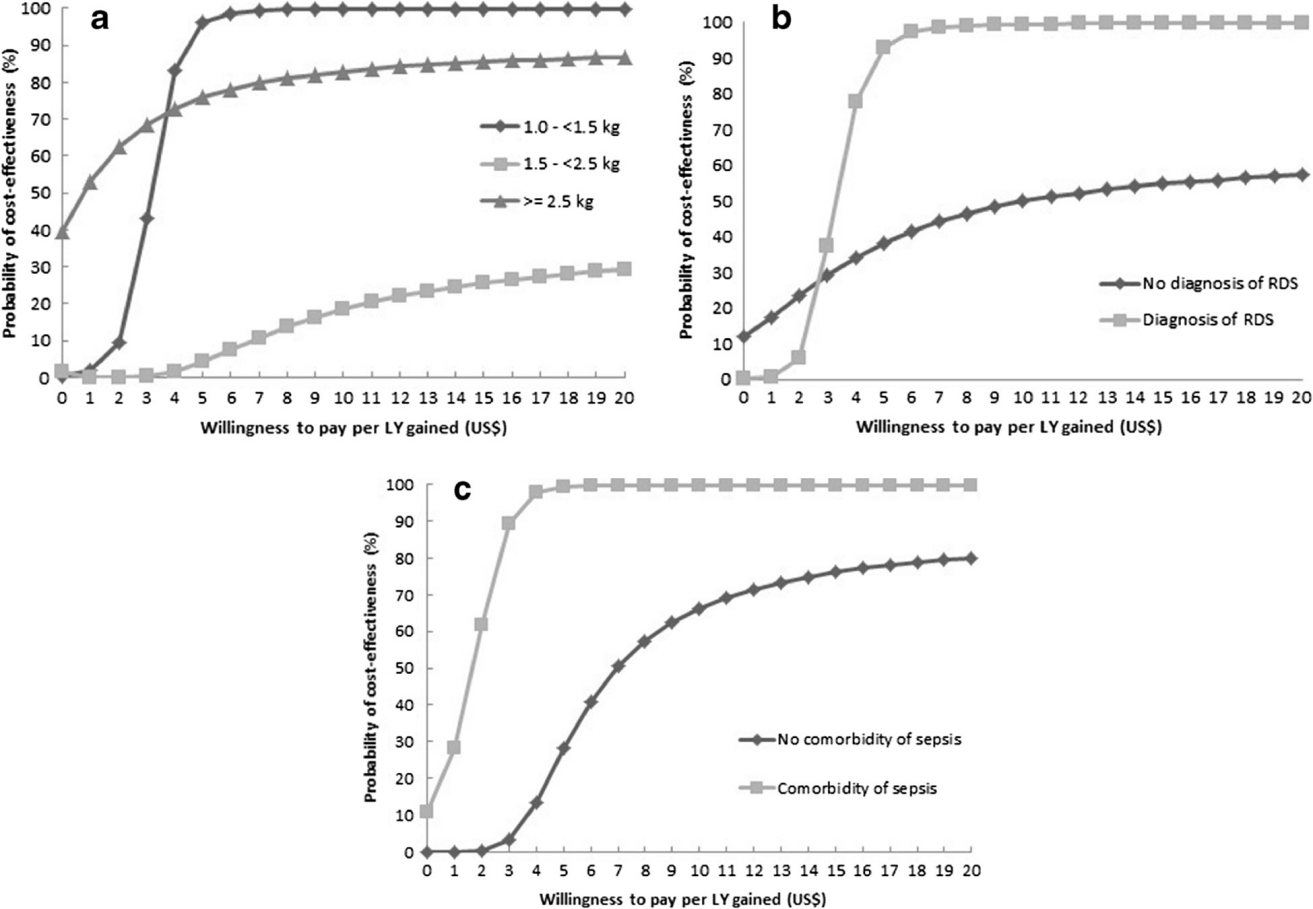
**Cost-effectiveness acceptability curve by the subgroups: a. birth weight, b. diagnosis of respiratory distress syndrome, and c. comorbidity of sepsis.**

The cost-effectiveness acceptability curves determine the probability that an intervention is cost-effective at a number of λ values. Overall, the probability that bCPAP was cost-effective was almost 100% at λ = US$20 (Figure 1). The probability of cost-effectiveness of bCPAP for patients with weight 1– < 1.5 kg (Figure 2a), a diagnosis of RDS (Figure 2b), or comorbidity of sepsis (Figure 2c) was higher than the probability of cost-effectiveness for patients with birth weight >2.5 kg or who had no diagnosis of RDS or no comorbidity of sepsis. Given the relatively small hospital bed-day cost, the incremental cost-effectiveness ratio for bCPAP compared to nasal oxygen did not significantly change (results not shown).

## Discussion

Using bCPAP is a highly cost-effective strategy in providing ventilatory support for neonates in Malawi. In our subgroup analysis, we determined that a patient’s birth weight, a diagnosis of RDS, and comorbidity of sepsis had a high impact on the cost-effectiveness of bCPAP. According to WHO international guidelines, interventions are considered highly cost-effective when the ICER in terms of cost per DALY is less than a country’s per-capita gross domestic product (GDP), cost-effective when the ICER is between one and three times the per-capita GDP, and not cost-effective when the ICER is above three times the GDP [19]. The national per-capita GDP of Malawi in 2012 was approximately US$268 [20]. Given that the ICER for bCPAP versus nasal oxygen is US$4.20 per LY gained, the bCPAP would be considered highly cost-effective by the international standards (assuming that the cost-effectiveness thresholds can be extended from DALYs to LYs).

There are several limitations to our study. First, the baseline efficacy data were obtained from a very small non-randomized population, with only 62 bCPAP and 25 nasal oxygen patients. The results of this study, including the subgroup analysis, should be considered preliminary and should be reexamined with data from a larger clinical trial. For ethical reasons, it is challenging to carry out a randomized controlled trial of potentially life-saving appropriate technologies when the benefits of counterpart technologies designed for high-resource settings are significant and well-documented. On the other hand, it is critical to assess technology performance in low-resource settings because performance is dependent on many aspects of infrastructure, including low staffing levels, potential interruptions in electrical power, uncontrolled climate, etc.

Currently, a prospective study evaluating the effectiveness of bCPAP compared to nasal oxygen therapy is being conducted at four central and 27 district hospitals in Malawi. Results of that study are expected after 2015.

Second, we used life tables for Malawi to extrapolate long-term effectiveness by translating clinical outcome in terms of 60-day survival to reflect discounted life expectancy. We assumed that once patients were discharged, they completed their lives following the standard life expectancy patterns. Although other exogenous factors can affect life expectancy, our analysis did not account for such factors.

Third, the overall calculated costs of treatment were largely based on the WHO-CHOICE estimates. These calculated costs were determined using an econometric model that used variables like GDP per capita and occupancy rate to predict country-specific hospital costs. However, these costs may not be a true representation of the actual bed-day costs in Malawi.

Fourth, we recognize that treatment with nasal oxygen at high concentration can reduce quality of life. There are limited data that address this issue that can be incorporated in an economic evaluation [21–23]. However, if we were able to include quality of life into the analysis then our conclusion would be strengthened because quality of life after nasal oxygen would likely be no better than quality of life after bCPAP making the incremental cost-effectiveness ratio for the bCPAP strategy even lower.

Despite the limitations, our calculations show that low-cost bCPAP is substantially below the international thresholds for being highly cost-effective. Even if the ICER was ten times its current value, bCPAP intervention would still be considered cost-effective. The increase in 60-day survival rates from nasal oxygen to bCPAP, the low cost of the bCPAP machine and accompanying equipment, the lack of noticeable complications from bCPAP, and the fact that this intervention takes place so early in life contribute to the extreme cost-effectiveness of bCPAP therapy.

Although from an economic standpoint, bCPAP is clearly cost-effective, we must recognize the infrastructural and cultural barriers to implementing the device on a national scale. While we chose not to include differences in labor demand, the administration of bCPAP therapy is an additional burden on the nurses who are already dealing with understaffed wards and high patient volumes. In addition, most clinicians are not trained to recognize the clinical indication for bCPAP, nor are they trained to administer it. Efforts are being made to train clinicians and incorporate bCPAP into the nursing curriculum in Malawi; however, transitioning bCPAP treatment to be a part of routine care has more obstacles than just cost. It requires the availability of a constant supply of equipment, trained clinicians, and a medical system that supports the administration of bCPAP. Cultural barriers also exist; for example, many Malawians associate nasal prongs with dying patients [24]. It takes extra time and effort to build the mothers’ trust that the nasal prongs used with bCPAP, or with nasal oxygen, will increase their children’s likelihood of survival.

## Conclusion

Our analysis indicates that this low-cost bCPAP is a highly cost-effective use of healthcare resources in Malawi. This intervention provides life-saving treatment at the earliest stages of life for a minimal cost, and offers an important strategy for the treatment of neonates with respiratory difficulty in other developing countries. We look forward to the results of the larger multicenter trial, which we expect will reinforce our conclusions of the effectiveness and cost-effectiveness of this intervention for neonates.

## Abbreviations

bCPAP: Bubble continuous positive airway pressure
RDS: Respiratory distress syndrome
INB: Incremental net benefit
LY: Life year
CI: Confidence interval
SD: Standard deviation
ICER: Incremental cost-effectiveness ratio
WHO: World Health Organization
GDP: Gross domestic product
NMB: Net monetary benefit
